# Pb^2+^ Effects on Growth, Lipids, and Protein and DNA Profiles of the Thermophilic Bacterium *Thermus Thermophilus*

**DOI:** 10.3390/microorganisms4040045

**Published:** 2016-12-06

**Authors:** Barbara Nicolaus, Annarita Poli, Paola Di Donato, Ida Romano, Giusi Laezza, Alessia Gioiello, Sergio Ulgiati, Florinda Fratianni, Filomena Nazzaro, Pierangelo Orlando, Stefano Dumontet

**Affiliations:** 1 Council National Research (C.N.R), Institute of Biomolecular Chemistry (ICB), via Campi Flegrei 34, Pozzuoli 80078, Italy; apoli@icb.cnr.it (A.P.); paola.didonato@icb.cnr.it (P.D.D.); iromano@icb.cnr.it (I.R.); giusi-lae@libero.it (G.L.); alessia.gioiello@unina.it (A.G.); 2 Department of Sciences and Technologies Parthenope, University of Naples, Centro Direzionale-Isola C4, Naples 80143, Italy; sergio.ulgiati@uniparthenope.it (S.U.); stefano.dumontet@uniparthenope.it (S.D.); 3 Council National Research (C.N.R), Institute of Food Science (ISA), via Roma, 64, Avellino 83100, Italy; fratianni@isa.cnr.it (F.F.); mena@isa.cnr.it (F.N.); 4 Council National Research (C.N.R), Institute of Applied Sciences and Intelligent Systems (ISASI), via Campi Flegrei 34, Pozzuoli 80078, Italy; p.orlando@isasi.cnr.it

**Keywords:** Pb^2+^ contamination, lead toxicity, thermophilic bacteria, *Thermus thermophilus*, Pb^2+^ resistance, lipids, protein profile, DNA melting

## Abstract

Extremophiles are organisms able to thrive in extreme environmental conditions and some of them show the ability to survive high doses of heavy metals thanks to defensive mechanisms provided by primary and secondary metabolic products, i.e., extremolytes, lipids, and extremozymes. This is why there is a growing scientific and industrial interest in the use of thermophilic bacteria in a host of tasks, from the environmental detoxification of heavy metal to industrial activities, such as bio-machining and bio-metallurgy. In this work *Thermus thermophilus* was challenged against increasing Pb^2+^ concentrations spanning from 0 to 300 ppm in order to ascertain the sensitiveness of this bacteria to the Pb environmental pollution and to give an insight on its heavy metal resistance mechanisms. Analysis of growth parameters, enzyme activities, protein profiles, and lipid membrane modifications were carried out. In addition, genotyping analysis of bacteria grown in the presence of Pb^2+^, using random amplified polymorphic DNA-PCR and DNA melting evaluation, were also performed. A better knowledge of the response of thermophilic bacteria to the different pollutants, as heavy metals, is necessary for optimizing their use in remediation or decontamination processes.

## 1. Introduction

Pb is a known human, animal, and environmental toxic metal. It occurs naturally in the Earth’s crust as Pb compounds and it is characterized by technological properties as high density, ductility, malleability, poor electrical conductivity, high corrosion resistance, and a low melting point [1,2,3,4]. It has been largely mined and used since the pre-industrial period, from late antiquity to the Middles Ages [5]. The technological properties of Pb brought to an early intensive exploitation of this metal causing a global contamination, which started as far as two millennia ago [6]. During the industrial period the use of Pb increased considerably, along with its environmental pollution and human toxicity, due to the high quantity of this metal added, inadvertently or through improper waste disposal, to water, soil and air [7].

Several human activities, such as smelters, war zones, and military firing ranges, transportation, and a host of industrial processes are direct sources of Pb environmental contamination [8]. In addition, many different products containing Pb, such as gasoline, cosmetics, water pipes, painting, and car batteries, heavily contribute to the global lead environmental contamination [1].

Such a world-wide diffused Pb pollution is causing a global health problem. Pb^2+^ poisoning, among other negative effects, impairs heme synthesis, causes detrimental consequences on central and peripheral nervous system (including negative behavioural and intellective effects), lowers nerve conduction velocity, and damages spermatogenesis and foetal development on humans [5].

The negative consequences of Pb pollution are not confined to health effects on humans. A number of scientific works assessed the ecotoxicological behaviours of this metal on wildlife of aquatic and terrestrial ecosystems, as reviewed by [4] and [9]. Pb can easily enter the terrestrial food chain via plant uptake, leading to the biomagnification of Pb in animal tissues, with the possible contamination of the human food chain [10,11]. The toxic effect of Pb^2+^ on the biomass and biochemical activities of soil microorganisms could impair the nutrient cycling in soil, threatening, together with number of other organic and inorganic pollutants, the global ecosystemic equilibrium [12].

The bacterial resistance to heavy metals is a topic of increasing interest from different scientific standpoints, including basic research, bio-remediation and bio-decontamination processes [13], and the development of bio-sensors [14]. As reviewed by Valls and De Lorenzo [13], the prokaryotic mechanisms of heavy metals resistance span from intra- or extracellular binding of the metal (useful for metal immobilisation) through a reaction with a methallothionein or by matching with an anion, the biotransformation of the toxic ion into a less toxic or a volatile form (as the case of Hg^2+^ transformed in methyl mercury) [15], and the use of metals as a final electron acceptor. The Gram-negative bacteria *Ralstonia* sp. CH_34_, a prokaryote able to thrive in millimolar concentrations of toxic heavy metals, uses a detoxifying mechanism that boosts the cell ion efflux systems, reducing that way the intracellular concentration of metals by active export [16].

There is a growing interest in the study of heavy metal detoxifications by thermophilic bacteria. Özdemir et al. [17] studied the passive mechanism of absorption of Cd^2+^, Cu^2+^, Co^2+^, and Mn^2+^ on the cell surface of thermophilic bacteria *Geobacillus thermantarcticus* and *Anoxybacillus amylolyticus*. The works of Hetzer et al. [18], Burnett et al. [19], and Chatterjee et al. [20] dealt with the absorption of Cd and other heavy metals on the cell surface of different thermophilic bacteria. Similarly, Babak et al. [21] studied the biosorption capacity of copper, lead, and zinc by *Geobacillus thermodenitrificans* and *Geobacillus thermocatenulatus*. Spada et al. [22] found a specific soluble, cytoplasmic metals binding protein in the thermophile *Thermus thermophilus* able to increase the cellular efflux of heavy metals like Zn, Co, and Cd.

All of these studies pointed out the possible role of thermophilic bacteria in heavy metal remediation, and also when sites to be depolluted experience harsh environmental conditions [23,24].

In these lines, we studied here the effects of Pb on the growth kinetic characteristics of the thermophilic bacteria *Thermus thermophilus* in order to give an insight on its Pb^2+^ resistance patterns by challenging it against increasing metal concentrations spanning from 0 to 300 ppm. Analysis of enzyme activities, protein profiles and lipid membrane modifications were carried out. In addition genotyping analysis of this bacteria growing in presence of Pb^2+^, using random amplified polymorphic DNA-PCR and DNA melting evaluation, were also performed.

## 2. Materials and Methods

### 2.1. Chemicals

Lead was used as a nitrate salt Pb(NO_3_)_2_ (Sigma-Aldrich, Milan, Italy) were prepared using reagent-grade water, sterilized by filtration, and were kept at 25 °C.

### 2.2. Biological System and Cultural Condition

*Thermus thermophilus* strain Samu-SA1 (DSM 15284, ATTC BAA-951) was isolated from the Mount Grillo (Baia, Naples, Italy) hot springs. It was grown at 75 °C in a 2 L bioreactor (Biostat-D, Bangalore, India) using 1 liter of medium (TH) containing peptone (Oxoid, Hampshire, UK) 8.0 g·L^−1^, yeast extract (Oxoid) 4.0 g·L^−1^, NaCl 2.0 g·L^−1^ at pH 7.0 [25]. The logarithmic phase of growth was at 24 h and the stationary phase of growth was at 48 h of incubation.

Pre-cultures were grown overnight in 500 mL shake-flasks filled with 200 mL of growth media at 75 °C in a shaking water bath at 300 rpm. 200 mL of pre-cultures were transferred into a shacked pilot bioreactor (BIOSTAT, Bangalore, India ) filled with 1000 mL of fresh media and incubated for two days at 75 °C. The cultures were maintained under an air flux of 20 mL·min^−1^·L^−1^. Solutions of Pb(NO_3_)_2_ were added to fresh media at concentrations of 100, 200, and 300 ppm of Pb^2+^. DNA and membrane lipids were extracted from bacterial cells, grown in presence of 100 ppm of Pb^2+^ and harvested after 3, 6, and 24 h. *T. thermophilus* cells, grown without adding Pb^2+^ and collected after 24 h of incubation, were used as control. The bacterial growth was measured spectrophotometrically at *λ* 540 nm, using a UV-VIS Spectrophotometer (Beckman, Brea, CA, USA).

The possible Pb bacterial precipitation was observed in 3000 mL shake-flasks filled with 1000 mL of growth media in which 200 mL of *T. thermophilus* pre-cultures were transferred. The flasks were incubated at 75 °C in a shaking water bath at 300 rpm for 48 h. The flasks contained: (a) a *T. thermophilus* pre-culture without Pb; (b) a *T. thermophilus* pre-culture spiked with 300 ppm of Pb(NO_3_)_2_; and (c) a sterile medium containing 300 ppm of Pb(NO_3_)_2_.

### 2.3. Homogenate Preparation

Cells of *T. thermophilus* were collected after 48 h of growth during stationary phase, both at 0 and 100 ppm Pb^2+^ by centrifugation at 15,000× *g* for 30 min. Wet cells (about 2 g) were lyophilized (Heto Dry Winner, Waltham, MA, USA), suspended (1:3 *w*/*v*) in 20 mM Tris–HCl (Applichem, Carlo Erba, Milan, Italy) at pH 8.0, lysed by the combined effect of ultrasonic treatment (Heat System Instrument, Waltham, MA, USA) (30 min) and lysozyme (Sigma) (6 mg of lysozyme 0.3 g^−1^ dry cells), and centrifuged at 25,000× *g* for 20 min. Protein content, enzymatic activities, and protein electrophoretic analysis were measured both on the supernatant of control samples (crude homogenate (CHT)-crude homogenate of *T. thermophilus*) and the samples spiked with 100 ppm Pb^2+^ (CHT + Pb^2+^-crude homogenate of *T. thermophilus* + Pb^2+^).

### 2.4. Lipid and Fatty Acid Analysis

Lipids from freeze drying cells, grown in optimal standard condition with and without Pb^2+^, collected after 48 h of incubation, were extracted using a CHCl_3_:CH_3_OH:H_2_O solution (65:25:4, by volume) and analysed by thin layer chromatography (TLC) on silica gel (0.25 mm, F_254_, Merck, Milan, Italy) eluted with the same solvent system. Total polar lipids were detected by spraying the plates with 0.1% Ce(SO_4_)_2_ (Sigma) in 2N H_2_SO_4_ followed by heating at 100 °C for 5 min. Phospholipids and amino-lipids were detected on the plates upon spraying with the Dittmer-Lester and the ninhydrin reagents, respectively, and glycolipids were visualized with α-naphtol [26]. Fatty acid methyl esters were obtained from complex lipids by acid methanolysis. Fatty acid methyl esters were detected using GC-MS Helwett-Packard 5890A instrument (Packard, San Diego, CA, USA), fitted with FID detector, and equipped with an HP-V column with a flow-rate of 45 mL·min^−1^ first at 120 °C for 1 min, and then increasing the temperature from 120 °C to 250 °C at a rate of 2 °C min^−1^ [27].

### 2.5. Extracellular Phase Preparation

Ammonium sulphate was added to the cell-free growing media (1 L) up to 80% of saturation. The precipitate was recovered by centrifugation (15,000× *g*, 1 h, 4 °C), dissolved in 20 mM phosphate buffer (pH 7.0), and dialysed (cut from 12,000–14,000 MW) (Medicell International Ltd., London, UK). The samples obtained (ET- extracellular phase of *T. thermophilus*) and (ET (extracellular protein phase) + Pb^2+^-extracellular phase of *T. thermophilus* + Pb^2+^) were used for protein determinations and electrophoretic analysis.

### 2.6. Enzyme Activities

β-glucosidase and α-maltosidase assays were based on the release of *p*-nitrophenol from the substrates *p*-nitrophenyl-β-d-glucopiranoside (Sigma) and 4-nitrophenyl-α-d-maltoside (Sigma, Milan, Italy), respectively. CHT was incubated at 75 °C with 0.1 mL of 20 mM substrate, and 0.8 mL of 50 mM sodium phosphate buffer at pH 7.0 for 5 min. The reaction was stopped by adding 1 mL of 1 M sodium carbonate and 3 mL of H_2_O. A blank containing 1 mL of buffer plus substrate was used to correct the thermal hydrolysis of the substrate. The absorbance of released *p*-nitrophenol was measured at *λ* 420 nm. One enzymatic unit of β-glucosidase and α-maltosidase was defined as the activity releasing 1µ mole of *p*-nitrophenol in 1 min from the specific substrates [28].

### 2.7. Protein Determination

The protein concentration was determined by the method of Bradford [29] using the Bio-Rad protein assay kit (BIO-RAD, Segrate, Milan, Italy) with bovine serum albumin as a standard.

### 2.8. Protein Profile

Fifty micrograms of CHT were analysed on 10% SDS-PAGE. Protein bands in the gel were visualized after staining with BioSafe Coomassie Blue (Bio-Rad) and subsequently destained in 9% acetic acid and 5% methanol. Pharmacia low molecular-weight electrophoretic standards were used as molecular weight markers [30].

### 2.9. Two-Dimensional Electrophoresis

Protein extracts were dissolved in 2.5 mL of an isoelectric focusing (IEF) buffer (8 M urea, 2 M thiourea, 5 mM DTT, 4% (*w*/*v*) Chaps, and 2% (*v*/*v*) Bio-Lyte 3/10 Ampholyte, pH 3–10, Bio-Rad), applied to a MicroRotofor (Bio-Rad Laboratories, Milano, Italy), and electrophoresed for 3 h at a constant power of 1 W at 10 °C [31]. After electrophoresis, 250 µL of each compartment were harvested; 750 µL of cold acetone was added, and fractions were incubated for 3 h at 4 °C. Samples were centrifuged at 8600× *g* for 10 min at 4 °C, then the supernatant was carefully removed. Pellets were gently air-dried, re-suspended in 50 µL of sterile deionised water and analysed by microcapillary electrophoresis on chip. An aliquot of 4 μL of each sample was mixed with 2 μL of the Protein 260 LabChip denaturing solution (Bio-Rad Laboratories, Milano, Italy) supplemented with 1 μL of β-mercaptoethanol (Sigma-Fluka, Milano, Italy) and heavy and light protein markers (Bio-Rad Laboratories, Milano, Italy). Samples were incubated at 100 °C for 3 min and mixed with 84 μL of ultrapure water. Molecular weight markers were prepared according to the Protein 260 Assay protocol (Bio-Rad Laboratories, Milano, Italy) and treated as described above. Separation and detection of proteins by molecular size were performed with the Experion apparatus (Bio-Rad Laboratories, Milano, Italy) over a range of molecular weights from 1.2 to 260 kDa (Experion™ Pro260 Analysis Kit; Bio-Rad Laboratories, Milano, Italy) using fluorescence detection and a 10-mW semiconductor laser at 630 nm. The data were analysed using the Experion software ((BIO-RAD, Segrate, Milan, Italy).

### 2.10. DNA Preparation and Genotyping Analysis

DNA was extracted and purified from about 250 mg of freeze-drying bacterial cells using the Genomic-DNA-Buffer Set and the Genomic-tip-100/G columns (QIAGEN SpA, Milano, Italy), according to manufacturer’s instructions. DNA concentration and molecular size were evaluated as previously described [32].

DNA melting temperature was evaluated as described by [27]. Briefly, DNA samples (about 45 kbp, as evaluated by agarose gel comparison to Lambda DNA) were subjected to thermal denaturation in a reaction mixture (final volume of 25 µL) containing 10 mM TRIS pH 8, 1 mM EDTA (ethylenediamine tetra-acetic acid), 20 mM NaCl, 1 × fluorescent DNA-intercalating dye EVA-green (Ethyl vinyl acetate) (Biotium, Fremont, CA, USA), and 200 ng of DNA, by using an iQ5 (Bio-Rad) PCR real-time apparatus. Determinations were performed in quadruplicate in a 96-well plate sealed by an optical tape (Bio-Rad). Well factors were obtained from a replicate plate containing the mixture without DNA, while the experimental plate was inserted in the iQ5 apparatus during a hold step at 37 °C. The incubation at 37 °C was resumed for a further 20 min, followed by a melting protocol from 50 °C to 100 °C in step of 0.2 °C, a dwell time of 15 s, and the acquisition of fluorescence data for each step. The fluorescence data were analysed by the iQ5 software and exported to the “Melting Profiler’’ version 0.7 software (Bio-Rad) for the evaluation of T_m_.

Random amplified polymorphic DNA-PCR (Random Amplification of Polymorphic DNA-Polymerase Chain Reaction, RAPD-PCR) assay was used to produce fingerprint patterns of *T. thermophilus* by using OPR-2, OPR-13 primers as described by [32]. (GTG)_5_ primer fingerprint analysis was performed according to Ronimus et al. [33]. PCR products were analysed by electrophoresis on microchip by using the DNA 7500 kit (Agilent, Santa Clara, CA, USA) and a 2100-Bioanalyzer equipped with 2100 EXPERT software Agilent, following the manufacturer’s instructions.

### 2.11. Statistical Analysis

Data, expressed as the means of three experiments ± standard deviation (SD), were analysed by one-way analysis of variance (ANOVA) and the Student-Newman-Keuls test, and differences were considered statistically significant for *p*-value < 0.05.

## 3. Results

### 3.1. Pb^2+^ Effects on Growth

**Figure 1:**
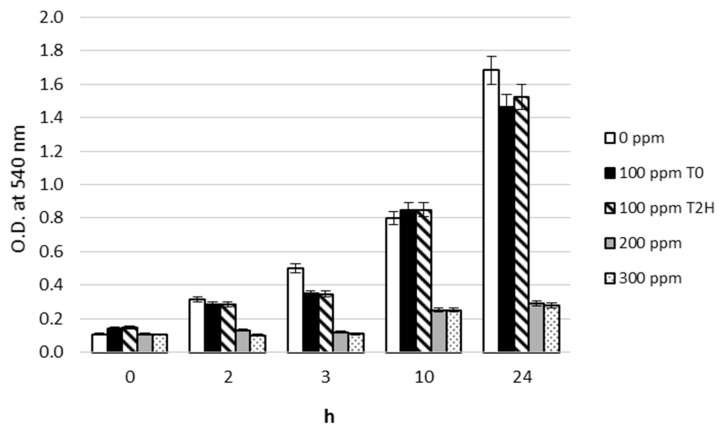
Lead effects on *Thermus thermophilus* growth. The microorganism was grown on media containing: 100 ppm, 200 ppm and 300 ppm of Pb^2+^, at different growth times. T_0_ = Pb^2+^ added at the beginning of incubation; T_2h_ = Pb^2+^ added 2 h after the beginning of incubation.

In Figure 1, the growth of *T. thermophilus* at 0, 100, 200, and 300 ppm of Pb^2+^ is shown. A marked negative effect of Pb^2+^ on growth was only observed at 200 and 300 ppm. The growth at these Pb^2+^ concentrations did not differ from each other along the incubation time. The mean values of absorbance (*λ* 540 nm) measured at 200 and 300 ppm of Pb^2+^ were 0.17 (±0.01), 0.19 (±0.01), 0.25 (±0.01), and 0.35 (±0.01) after 2, 3, 10 and 24 h of incubation, respectively. The Pb^2+^ at concentration of 100 ppm, either added at T_0_ or at T_2h_, did not show any significant difference from the control. The mean values of absorbance were 0.31 (±0.02), 0.43 (±0.01), 0.85 (±0.01), and 1.58 (±0.11) after 2, 3, 10, and 24 h of incubation, respectively.

**Figure 2:**
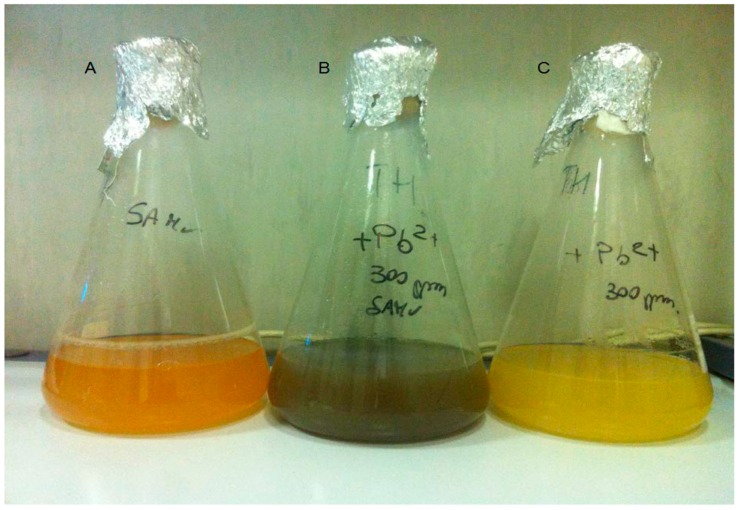
*T. thermophilus* growth at 75 °C with 300 ppm of Pb^2+^ and without presence of Pb after 48 h of incubation. (**A**) Cells growing in TH medium without Pb^2+^; (**B**) cells growing in TH medium (as described in material and methods) + 300 ppm of Pb^2+^; and (**C**) sterile medium TH + 300 ppm of Pb^2+^.

Figure 2 shows the precipitation of metallic Pb by *T. thermophilus*. In flask B, inoculated with bacterial pre-culture spiked with 300 ppm of Pb(NO_3_)_2_, a black precipitate is formed after 48 h of incubation, suggesting a possible oxidation of Pb^2+^ to Pb.

### 3.2. Pb^2+^ Effects on Lipid Pattern and Fatty Acid Composition

**Figure 3:**
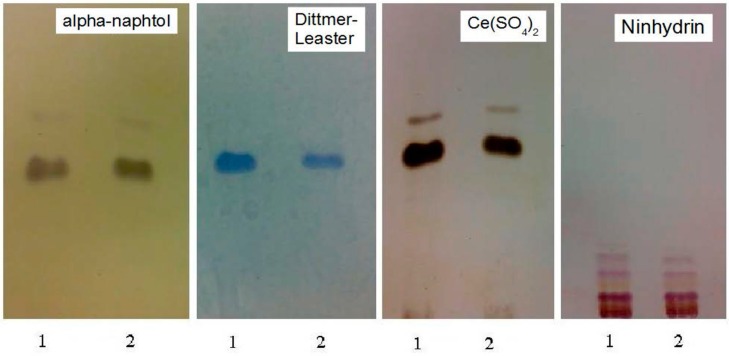
Thin layer chromatography (TLC) of polar lipids, extracted from freeze drying cells of *Thermus thermophilus* strain Samu-SA1 grown in standard conditions (lanes 1) and with lead addition (lanes 2). Elution: CHCl_3_:CH_3_OH:H_2_O (65:25:4, by vol.). The lipid pattern was detected on the plates upon spraying with alpha-naphtol, the Dittmer-Lester, Ce(SO_4_)_2_ and ninhydrin reagents.

The basal lipid pattern of *T. thermophilus* grown in 100 ppm of Pb^2+^ added at beginning of the incubation (T_0_) was compared to that obtained in the absence of lead. TLC analysis of lipid fraction suggested that lead mainly affected the polar lipids since a decrease of phospholipid abundance was evidenced (Figure 3, panel Dittmer Leaster). This result is of particular interest as phospholipids are the main taxonomic markers for the genus *Thermus.*

The results of fatty acid methyl ester (FAME) are reported in Table 1. In the sample exposed to 100 ppm of Pb^2+^ the percentages of both *iso*-C15:0 and *anteiso*-C17:0 were lower than the control. These saturated fatty acids was paralleled by an enhanced levels of *iso*-C17:0, which was 12.38% higher than the control.

### 3.3. Pb^2+^ Effects on Cell Protein Profiles and Enzyme Activities

Table 2 shows the effect on protein content of the crude homogenate and extracellular phase of *T. thermophilus* grown in 100 ppm of Pb^2+^ added at the beginning of the incubation. Pb^2+^ reduced the protein content by 13% in crude homogenate and by 14.5% in extracellular phase.

*T. thermophilus* synthesizes a considerable array of hydrolytic enzymes [25]. Among them, β-glucosidase and α-maltosidase are particularly important as they allow the utilization of a wide variety of carbon sources offering a survival edge in case of limiting nutritional conditions [34]. Pb^2+^ reduces the activity of these enzymes by 9.7% and 17.5%, respectively, as compared to the control.

**Figure 4:**
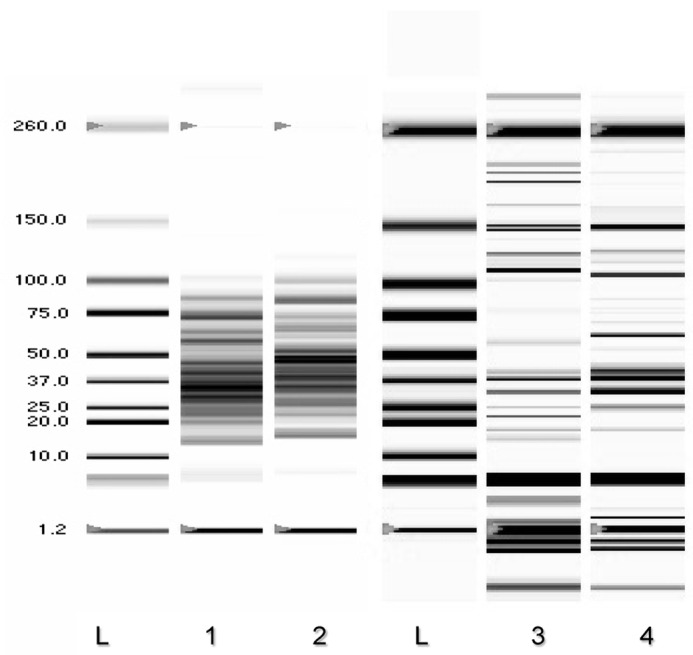
Capillary microelectrophoresis on specific microchip of crude homogenate (CHT) and extracellular protein phase (ET) of *T. thermophilus* grown in 0 and 100 ppm of Pb^2+^. L = Ladder standards; (**1**) CHT: crude homogenate of *T. thermophilus*; (**2**) CHT + 100 ppm Pb^2+^: crude homogenate of *T. thermophilus* added at fermentation start time; (**3**) ET: extracellular phase of *T. thermophilus*; (**4**) ET *+* 100 ppm Pb^2+^: extracellular phase of *T. thermophilus* added at the beginning of the incubation.

Protein profiles of CHT and CHT + Pb^2^^+^, and ET and ET + Pb^2^^+^ are shown in Figure 4, lanes 1–4. They ranged between 18 kDa to 118 kDa. In the first portion of the gel (up to 30 s of migration, showing low MW proteins), CHT exhibited four proteins of 14.31 kDa, 20.01 kDa, 22.95 kDa, and 24.79 kDa; CHT + Pb^2+^ showed three proteins of 16.12 kDa, 22.87 kDa, and 26.94 kDa in the corresponding area of gel. In the subsequent portion of the gel (30–35 s of migration), CHT showed six distinctive proteins, at 30.26 kDa, 34.17 kDa, 36.71 kDa, 41.26 kDa, 46.09 kDa, and 53.23 kDa; therefore, CHT + Pb^2+^ exhibited seven proteins, some of which with the same MW of the control (34.20 kDa, 41.23 kDa, 46.06 kDa, and 52.56 kDa) and three with different MW (39.28 kDa, 43.30 kDa and 49.20 kDa). The last portion of the gel showed proteins with a higher MW; in particular, CHT showed five proteins at 58.67 kDa, 63.82 kDa, 68.08 kDa, 72.30 kDa, and 86.53 kDa; these last three were also found in CHT + Pb^2+^. Furthermore, CHT + Pb^2+^ showed proteins, with a presumptive MW of 61.48 kDa, 64.99 kDa, and 99.08 kDa, which were lacking in the control. The presence of Pb affected also the secretion of different proteins in the extracellular environment, with MW ranging between 15 and 200–236 kDa (Figure 4, lanes 3–4); some proteins of 20 kDa, 24.6 kDa, 112 kDa, and 146 kDa were detected only in ET; therefore ET + Pb^2+^ showed three other specific proteins of 54.54 kDa, 72.11 kDa, and 88.12 kDa.

### 3.4. Pb^2+^ Effects on Protein Pattern; Two-Dimensional Electrophoresis

**Figure 5:**
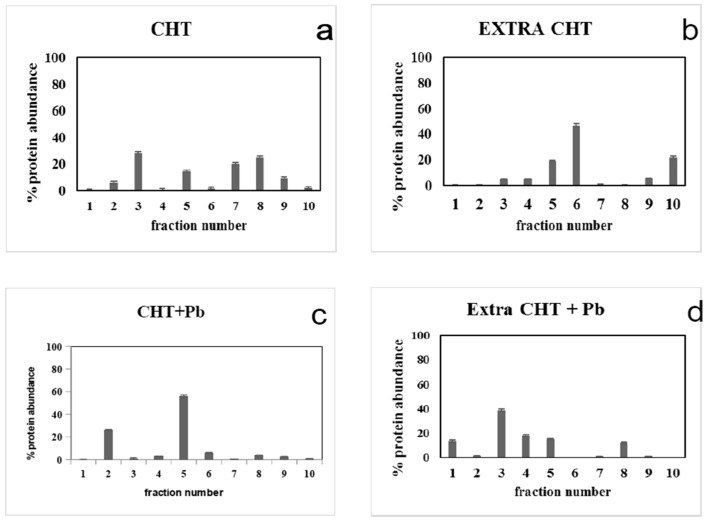
Histograms of protein distribution after micro 2D electrophoresis of crude homogenate (CHT and CHT+ Pb^2+^, panels (**a**) and (**c**), respectively) and extracellular proteins (Extra CHT and Extra CHT + Pb^2+^, panels (**b**) and (**d**), respectively) of *T. thermophilus*. On x axis are indicated the number of fractions. Y axis indicates the percentage. Each sample was tested in triplicate.

Proteins of CHT, ET, CHT + Pb^2+^ and ET + Pb^2+^ were analysed by micro two dimensional electrophoresis. In the first dimension, proteins were separated into 10 liquid microfractions according their isoelectric point (pI). Each fraction was then analysed by electrophoresis on chip; such a system separated the proteins according to their molecular weight, allowing to also measure the concentration and the relative percentage of each protein for each fraction. Results are shown in Figure 5.

**Figure 6:**
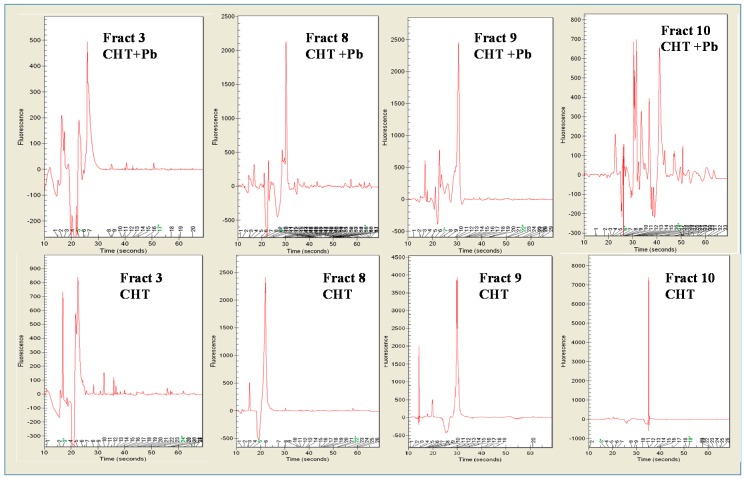

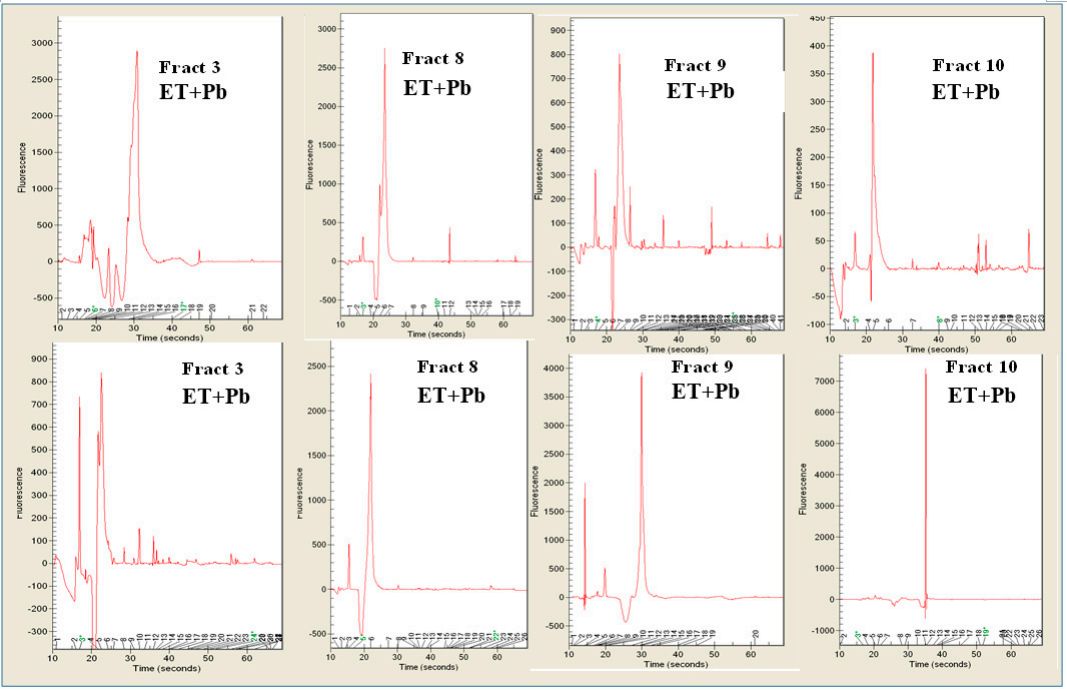
Electropherograms of some fractions obtained after micro 2D electrophoresis of the proteins present in *Thermus thermophilus* grown with (CHT + Pb^2+^) and (CHT) without Pb ^2+^ and excreted by the strain grown with (ET + Pb^2+^) and (ET) without Pb^2+^. Proteins were divided in ten fractions, following their pI.

Proteins of CHT were distributed among the 10 fractions obtained after isoelectric focusing (IEF); fraction 3 (pH 5.0, 27.57% of total) and mainly fractions 7–10 (with a pI from pH 8.0 to pH 10.1) contained the majority of the proteins (more than 85%) (Figure 5a). The presence of Pb^2+^ led to the synthesis of proteins mainly in the acidic-neutral Ip (Figure 5c): in particular in fraction 2 (pH 4.0, 26.09%) and mainly in fraction 5 (pH 7.0, 56.01%). Therefore, the two different conditions were capable of modifying the expression of the extracellular proteins (Figure 5b,d). In the usual condition of growth (Figure 5b), *T. thermophilus* fundamentally secreted proteins having neutral-alkaline pI, most of them were detected at pH 7.0 (fraction 5, 19.2% of the total proteins) and particularly at pH 7.5 (fraction 6, containing 46.94% of the total proteins). Another 21.54% of proteins was found at pH 10, thus, at an extremely alkaline pH. Extracellular proteins of bacteria grown in the presence of Pb^2+^ (Figure 5d) exhibited principally an acidic-neutral pI; in fact, they were found in fraction 1 (pH 2.5, 13.2%), and, in fraction 3, the most abundant (pH 5.0, containing 38.8% of the total proteins), decreasing conversely with the increase of pH (17.71% in fraction 4, at pH 6.0; 15.12 % in fraction 5, at pH 7.0) until 11.8% of the total extracellular proteins were found in the alkaline zone (fraction 8, pH 8.5). Pb limited the synthesis and the expression of some proteins, as shown by the comparative analysis of the singular fractions (Figure 6).

This could be observed, in particular, for fraction 3, and for fractions having an pI ranging from 8.0 to 10.1. Fraction 3 (pH 5.0) showed a very low amount of proteins, and the most abundant protein (16.9 kDa) represented 96% of the total protein amount in that fraction. On the contrary, the synthesis and expression of the proteins in the same fraction were at least five times higher. Herein, three proteins were particularly abundant, at 15.5 kDa, 21.7 kDa, and 33.2 kDa. Fractions with pI ranging from 8.0 to 10.1 contained those proteins which synthesis and expression were probably much more prejudiced by the presence of Pb, as shown by their electropherograms. Some of them are also represented as electropherograms (Figure 6). The behaviour was similar as regard as the production of the extracellular proteins. The differences were found in the acidic area and mainly in the neutral-alkaline area, represented by the range of fractions 7–10. In the acidic area, fraction 2, although exhibiting a similar profile, showed a different percentage of the same proteins: in fact, ET showed three peaks, at 20 kDa (the most abundant, 84%), 23 and 82 kDa, in ET + Pb^2+^, the most abundant peaks showed MW of 10 kDa (41%) and 27 kDa (27%). Peaks at MW 54 kDa, exhibited both in fraction 3 of ET and in ET + Pb^2+^ showed a completely different percentage (2.4% and 79%, respectively). Fraction 3 showed a peculiar peak, present only in the absence of Pb^2+^; in the neutral-alkaline area, fraction 7 exhibited a similar profile but proteins resulted present in different percentage; this is true, in particular, for a protein at 23 kDa, which presence of Pb increased its percentage (from 27% to 41%, respectively). Fraction 9 of ET showed two peaks at 16.5 and 24.4 kDa, which represented almost the totality of the proteins present in such fraction (91.3% and 4.9%, respectively): such proteins seemed absent in ET + Pb^2+^. In fraction 10, a protein at MW 91.05 kDa (88.6%) was detected in ET; the most abundant protein (83%) found in fraction 10 of sample ET + Pb^2+^ showed a higher MW (91 kDa).

The framework obtained from the analysis of proteins of *T. thermophilus* grown with or without Pb^2+^ leaves us some assumptions to be analysed in depth in future surveys. One of them could assume that the strain adapted itself to the presence of Pb producing more proteins in Ip acid-neutral area (Figure 5c), with respect to the control, that instead showed a more harmonious distribution, in terms of Ip, of its proteins; this was also plain with respect to the extracellular proteins, which showed a neutral-alkaline Ip in the control (with the exception of the fraction 10); the Ip distribution shifted toward from the acidic to the neutral area in the presence of Pb^2+^.

Fraction 6 could represent a particular case. In CHT + Pb^2+^, its concentration increased up to five times with respect to the control (Figure 5a,c, respectively); on the contrary, the amount of extracellular proteins expressed by the strain in the presence of Pb^2+^ was about eight times lower than those intracellular, and even 40 times lower if compared to the amount of extracellular proteins of the control. At this point, we could also hypothesize that the proteins present in such fractions could be normally produced and then expelled outside. In the presence of Pb^2+^ there is an accumulation of these proteins within the cell, which probably bind in some way the Pb^2+^ or, in some manner, counter its damaging action.

### 3.5. Determination of DNA Melting Temperature

Concentrations of 100 ppm Pb^2+^ increased the DNA melting temperature (*Tm*) of *T. thermophilus* cells, after 6 h of incubation, of 1.4 °C as compared to the control, in particular from *Tm* = 89 °C to *Tm* = 90.4 °C. Such *Tm* increase theoretically should correspond to a 3% increase of guanine and cytosine in the DNA, but it could also be the result of stabilization of DNA domains conferring a bit more stability to denaturation. No further increase was observed in cells incubated for 24 h. These data suggest an epigenetic modification in a cluster of genes related to heavy metal resistance.

### 3.6. Random Amplified Polymorphic DNA-PCR

**Figure 7:**
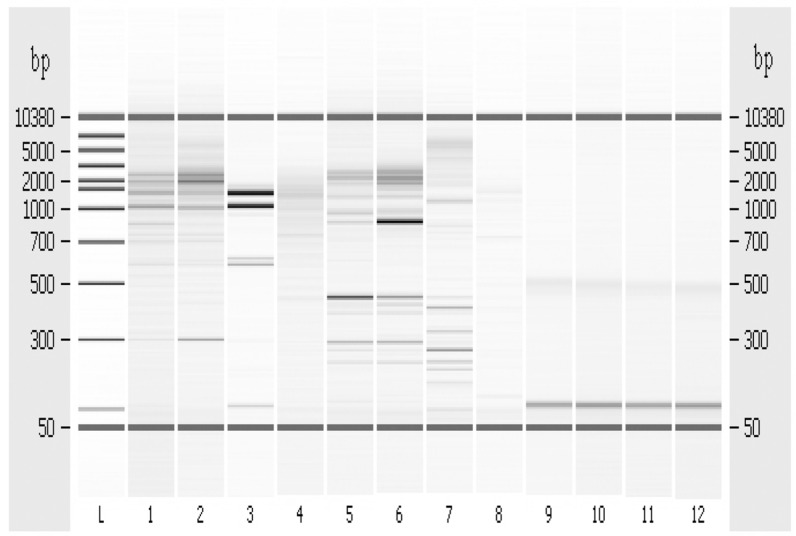
RAPD-PCR-fingerprint. L = Ladder standards. Lanes 1–4, Amplification with OPR2 primer (5^1^-CACAGCTGCC-3^1^). Lanes: (**1**) *T. thermophilus* (control); (**2**) *T. thermophilus* + 100 ppm Pb^2+^, collected after 3 h; (**3**) *T. thermophilus* + 100 ppm Pb^2+^, collected after 6 h; (**4**) Ctr negative (-DNA). Lanes 5–8, Amplification with OPR13 primer (5^1^-GGACGACAAG-3^1^). Lanes: (**5**) *T. thermophilus* (control); (**6**) *T. thermophilus* + 100 ppm Pb^2+^, collected after 3 h; (**7**) *T. thermophilus* + 100 ppm Pb^2+^, collected after 6 h; (**8**) Ctr negative (-DNA). Lanes 9–12, Amplification with (GTG)_5_ primer oligonucleotide. Lanes: (**9**) *T. thermophilus* (control); (**10**) *T. thermophilus* + 100 ppm Pb^2+^, collected after 3 h; (**11**) *T. thermophilus* + 100 ppm Pb^2+^, collected after 6 h; and (**12**) Ctr negative (-DNA).

This analysis was performed to confirm the hypothesis that the *Tm* variation could be correlated to possible genetic mutations. The results of RAPD-PCR of *T. thermophilus* DNA are shown in Figure 7.

The comparison between the control and the DNA extracted from cells incubated in presence of 100 ppm of Pb^2+^, collected at three and 6 h of growth, pointed out that Pb^2+^ exposure caused an evident change in the potential binding sites of both RAPD-primers used for this assay, suggesting the presence of possible point mutations. In particular, in the lane 3 of Figure 7 related to *T. thermophilus* cells, collected after 6 h of 100 ppm Pb^2+^ exposition (OPR-2), there is a DNA band of about 65-kbp which was lacking in the control (Figure 7, lane 1). In addition, we detected a very intense signal of about 48-kbp DNA, in the lane 5 of Figure 7, corresponding to control cells (OPR-13) only weakly detectable in Pb^2+^ treated cells. On the contrary (GTG)_5_ primer did not produce a relevant finger in both control and treated cell.

Therefore, both the DNA melting temperature and the random amplified polymorphic DNA-PCR results suggest that Pb^2+^ at concentration of 100 ppm is able to induce pinpoint mutations. RAPD confirmed the presence of point mutations.

## 4. Discussion

The use of thermophilic bacteria in heavy metal remediation of polluted sites, or for metal removal from industrial waste, has been recently explored by different authors [17,18,19,20,35]. The use of thermophilic bacteria as passive heavy metal accumulators depends on their heavy metal resistance, the degree of contamination and the physical and chemical characteristics of the matrix to be remediated or de-polluted. The known ability of such bacteria to withstand harsh environmental conditions could be of assistance in accommodating difficult physical and chemical settings of polluted matrices [36,37].

Our results point out a possible role of *T. thermophilus* in the precipitation of soluble and toxic Pb^2+^ into biologically inactive metallic Pb. This could be a newly-found detoxification mechanism of thermophilic bacteria, as the previously described ones were passive mechanisms of heavy metals absorption on the bacterial cell surface [17,18,19,20], or through a specific soluble, cytoplasmic metal-binding protein able to increase the cellular efflux of heavy metals [22].

The increase of T*m* during DNA melting could be explained by a potential increase of G + C content (that corresponds to point mutations AG or TC, transitions), but it could also be the result of the stabilization of DNA domains conferring more stability to denaturating agents. *T. thermophilus* seems to show a high plasticity, both genotypically and phenotypically, allowing adaptation to stressing environmental scenarios. Our strain seems capable of surviving at high Pb^2+^ concentrations in the culture media, both by diminishing its concentration (transforming the soluble Pb^2+^ into insoluble Pb, probably thanks to redox proteins) and by accommodating (probably through point mutations) to toxic metal concentrations modifying lipids, and protein and DNA profiles.

## 5. Conclusions

In conclusion, *T. thermophilus*, a harmless bacteria ubiquitous in soil, could be a good candidate for heavy metals removal from different contaminated matrices characterized by harsh environmental conditions. A better knowledge of the response of thermophilic bacteria to the different pollutants, as heavy metals, is necessary for optimizing their use in such a context.

## Figures and Tables

**Table 1:** Major fatty acid methyl ester profiles of *T. thermophilus* grown in 0 and 100 ppm Pb^2+^. Values are the mean of three experiments ± standard deviation (SD).

Fatty Acids	0 ppm Pb^2+^	100 ppm Pb^*2+*^
*iso*-C15:0	21.54 ± 1.1%	16.96 ± 0.9%
*iso*-C17:0	47.98 ± 2.2%	60.36 ± 3.1%
*anteiso*-C17:0	12.24 ± 0.4%	10.08 ± 0.3%

**Table 2:** Pb^2+^ effect on protein contents and enzyme specific activities of *T. thermophilus*. Values are the mean of three experiments ± SD.

Sample	Proteins in Crude Homogenate (mg/mL)	Proteins in Crude Homogenate (mg/g dry cell)	Proteins in Extracellular Fraction (mg/mL)	Proteins in Extracellular Fraction (mg/g dry cell)	β-Glucosidase Specific Activity (U/mg)	α-Maltosidase Specific Activity (U/mg)
0 ppm Pb^2+^	36.03 ± 1.8	51.47 ± 2.2	2.07 ± 0.06	2.96 ± 0.07	4.1 ± 0.12	8.0 ± 0.22
100 ppm Pb^2+^	31.29 ± 1.5	44.7 ± 1.9	1.77 ± 0.05	2.53 ± 0.06	3.7 ± 0.11	6.6 ± 0.18
